# Eye Movements Reveal Delayed Use of Construction-Based Pragmatic Information During Online Sentence Reading: A Case of Chinese *Lian*…*dou* Construction

**DOI:** 10.3389/fpsyg.2019.02211

**Published:** 2019-10-30

**Authors:** Chuanli Zang, Li Zhang, Manman Zhang, Xuejun Bai, Guoli Yan, Xiaoming Jiang, Zhewen He, Xiaolin Zhou

**Affiliations:** ^1^Academy of Psychology and Behaviour, Faculty of Psychology, Tianjin Normal University, Tianjin, China; ^2^School of Psychology, University of Central Lancashire, Preston, United Kingdom; ^3^Department of Psychology, Tongji University, Shanghai, China; ^4^School of Psychological and Cognitive Sciences, Peking University, Beijing, China; ^5^Beijing Key Laboratory of Behaviour and Mental Health, Beijing, China; ^6^Key Laboratory of Computational Linguistics (Ministry of Education), Peking University, Beijing, China; ^7^IDG/McGovern-PKU Institute of Brain Research, Peking University, Beijing, China; ^8^Institute of Linguistics, Shanghai International Studies University, Shanghai, China

**Keywords:** eye movements, sentence construction, pragmatic constraint, Chinese reading, pragmatic inference

## Abstract

An event-related potential (ERP) study demonstrated that construction-based pragmatic constraints in Chinese (e.g., *lian*…*dou* that constrains a low-likelihood event and is similar to *even* in English) can rapidly influence sentence comprehension and the mismatch of such constraints would lead to increased neural activity on the mismatching word. Here we examine to what extent readers’ eye movements can instantly reveal the difficulties of mismatching constraints when participants read sentences with the structure *lian* + *determiner phrase* + *object noun* + *subject noun* + *dou* + *verb phrase (VP)* + *final commenting clause*. By embedding high-likelihood or neutral events in the construction, we created incongruent and underspecified sentences and compared such sentences with congruent ones describing events of low expectedness. Relative to congruent sentences, the VP region of incongruent sentences showed no significant differences on first-pass reading time measures, but the total fixation duration was reliably longer. Moreover, readers made more regressions from the VP and the sentence-final region to previous regions in the incongruent than the congruent condition. These findings suggest that the effect of pragmatic constraints is observable during naturalistic sentence reading, reflecting the activation of the construction-based pragmatic information for the late integration of linguistic and extra-linguistic information at sentential level.

## Introduction

To make sense of linguistic inputs in different communicative contexts, readers need to incrementally build linguistic representations based on local semantic constraint, and integrate this local representation with extra-linguistic (e.g., pragmatic) information in real time (Zhou et al., 2009, 2010; Jiang and Zhou, 2012; Jiang et al., 2013a, b; Clifton et al., 2016). The negotiation of meanings derived at different representation levels determines when and how the pragmatic meaning is activated and used during sentence comprehension (Politzer-Ahles et al., 2013). In this sentence, *Even a rich person cannot afford such an expensive house*, a less likely event *a rich person cannot afford an expensive house* is constrained by the *even* construction, denoting the unexpectedness of what is described in the construction, and implying that any event which is more likely to happen than the embedded event must occur. If the event does not rank at the lowest end of the scale, embedding such event in the construction can result in infelicitousness (Fauconnier, 1975; Yuan, 2006). However, it remains unclear whether such construction-based pragmatic constraint can exert an immediate impact on local linguistic representation building and at what stage the detection of anomaly of such pragmatic constraint affects the relevant processes (Filik et al., 2009; Jiang et al., 2013a; Jiang and Zhou, 2014).

Extensive evidence from ERPs (event-related brain potentials) has suggested that readers can immediately detect when an upcoming word is pragmatically incongruent with the prior sentential/discourse/communicative context (such as the prediction generated from the discourse representation, reader’s world knowledge, or even the speaker identity), as indicated by an increased N400 response on the word that indexes an increased effort of integrating the word into the pragmatic context (e.g., Van Berkum et al., 1999, 2003, 2008; Hagoort et al., 2004; Jiang et al., 2013a, b; Nieuwland, 2013; Li et al., 2014). Some studies showed a relatively late starting (∼400 ms) but prolonged negativity effect on the words (e.g., sentence-initial scalar quantifiers *some kids were riding bicycles*) preceded by a context mismatching the pragmatic meaning of the quantifier (e.g., a picture showing all kids were riding bicycles). This negative response indexes a process of canceling or inhibiting initially built pragmatic representation, implicitly indicating that pragmatic information is instantly used for online sentence processing (Politzer-Ahles et al., 2013). In contrast, research using the eye-tracking technique has observed plenty inconsistent findings (e.g., Rayner et al., 2004; see also Warren, 2011 for a review). It is evident that ERP research typically adopts rapid serial visual presentation (RSVP) paradigm in which one word at a certain time is presented in the screen and participants are required to fixate the target and avoid making eye movements. Therefore, the word-by-word presentation prevents natural eye movement behavior that usually occurs during normal reading such as parafoveal processing (i.e., information about a word in the parafovea is available before the word is directly fixated), word skipping, refixation, and regression. In the present study we used the same stimuli from an ERP study conducted by Jiang et al. (2013a) and employed an eye movement tracking technique to examine the precise time course of processing Chinese construction-based pragmatic information during normal sentence reading.

Previous eye-tracking studies on pragmatic processing are inconclusive about how early the pragmatic constraints can impact the eye-movement measures during on-line sentence reading. The case of *pragmatic implausibility* (the use of language is still plausible if one’s world knowledge permits the language use in rare cases, similar to the label “pragmatic anomaly”) showed mixed evidence. For example, Murray and colleagues (Murray, 1998; Murray and Rowan, 1998; Kennedy et al., 2004) investigated whether readers could immediately detect the incongruence when a word was pragmatically incongruent with the context in a task where they were not explicitly reminded of the incongruent word in the sentence (i.e., when matching a probe sentence with the target sentence). Such incongruence arose given the low probabilistic expectancy of the linguistic input (the noun) in the given or inferred contextual information (the verb, e.g., *the savages/uranium smacked the child*). The authors reported very early *parafoveal-on-foveal* effects of pragmatic plausibility (although this effect appeared marginal in statistical significance), such that the pragmatically implausibility of the critical word inflated first pass reading times on its preceding regions (*uranium*), thus can be detected parafoveally before that word was directly fixated (see Drieghe, 2011; Clifton et al., 2016; for reviews).

The very early pragmatic effects reported in Murray and colleagues seem to be restricted to local adjacent linguistic combination, and such parafoveal effect disappeared when the noun and verb were separated by other adjunctive phrases (such as *the princess with blonde hair delivered the packages;* Murray, 2006). Ni et al. (1998) and Braze et al. (2002) used similar materials and asked participants to read sentences like *The wall will surely crack_*baseline*__/_bite_*pragmatic anomaly*_ after a few years in this harsh climate*. They found that participants immediately detected the anomaly just at the critical regions “*bite after*” relative to “*cracking after*,” whereas the pragmatic anomaly did not manifest its effect until the word after the verb “*bite.*” In Ni et al. (1998), pragmatic anomaly and baseline condition did not differ until the final region of the sentence (*this harsh climate)*, with the first pass reading time being increased for the former rather than for the latter condition. Furthermore, Rayner et al. (2004) investigated the time course of implausibility effect in the sentence frame (e.g., *John used a knife_*b*__*aseline*_/an axe_*i*__*mplausible*_ to chop the large carrots for dinner last night*), and found no effect of implausibility during first pass reading of *carrots*. The potential effect was further delayed in the condition in which the world-knowledge permits no way out (the impossible condition, e.g., *John used a pump to inflate the large carrots for dinner*). The *go-past* measure of eye movement, which includes the amount of time readers spent on the target word as well as the one spent on constituents preceding the target before moving forward to new portions of the sentence, was influenced by implausibility; but the effect size of this measure was fairly small, indicating that the impact of context has no immediate effect on eye movements during reading (see also Warren, 2011 for a review).

The pragmatic implausibility seems to be affected by the discourse-level contextual information (Ferguson and Sanford, 2008; Xu et al., 2017). For example, although there was no effect of pragmatic anomaly on the first fixation or other first-pass measures on the anomalous word (e.g., *pick up the lorry*), such an effect was found on the region following the anomalous word when the context indicated that the person who exerted the action was a cartoon or fictional character (Filik, 2008). Moreover, the effect of mismatch on one’s world knowledge was manifested as longer first-pass measures on words preceded by a real-world context (*Evolution dictates that cats are carnivores and cows are vegetarians. Families could feed their cat a bowl of carrots and it would gobble it down happily*) but as longer total reading time on words preceded by a counterfactual world context (*If cats were vegetarians they would be cheaper for owners to look after. Families could feed their cat a bowl of fish and it would gobble it down happily;* Ferguson and Sanford, 2008). Similar absence in early first-pass measures was also observed on words that were implausible over the clause connected by a concessive conjunction *(Grandma has moved from Shenyang to Hainan, although she liked the winter there being warm)*, but not on words preceded by a causal conjunction *(Grandma has moved from Hainan to Shenyang, although she liked the winter there being warm*; Xu et al., 2017).

Construction-based pragmatic constraints implemented by different focus particles also moderate different stages of processing during online reading. A relevant study (Filik et al., 2009) investigated how quickly readers integrate information against their world knowledge while interpreting incoming words that are constrained by sentence-initial focus particles, such as *only* or *even*. Linguistically, both *only* and *even* contrast focused elements with an alternative set of implicit elements, but each introduces different linguistic expectations. In the subsequent sentence following the particles, o*nly* constrains a highly likely event and excludes contextually relevant alternatives whereas *even* includes these alternatives and constrains an unexpected or surprising event. If linguistic information in the sentence does not match the pragmatic implication of *only* or *even* and the consequent interpretation does not match a reader’s expectations or world knowledge, it may generate incongruency and disrupt text comprehension. Participants read sentences like *Only/Even students taught by the best/worst teacher passed the examination in the summer*. The subsequent description of a less likely event in sentences beginning with *only* is incongruous (*Only students taught by the worst teacher passed the examination*), while a highly likely event within sentences that begins with *even* leads to incongruity (*Even students taught by the best teacher passed the examination*) with readers’ world knowledge. Filik et al. (2009) found that for sentences with *only*, there was longer first-pass reading time spent on the critical region (e.g., *passed the examination*) when information provided by the text became incongruous, indicating that the incongruence was detected fairly quickly. In contrast, for sentences with *even*, the effects of anomaly emerged with a longer delay, and were manifested only on the post-critical region (e.g., *in the summer*). Furthermore, readers were more likely to make regressions back to the text that contained a focus when sentences with *even* described a set of likely events.

Overall, the empirical evidence concerning the time-course of processing pragmatic constraints, established with the eye-tracking approach, is mixed and mostly restricted to alphabetic languages like English. Investigating this issue in a completely different orthography may provide further evidence regarding the time course of when readers detect pragmatic anomalies. Unlike English, Chinese orthography is logographic, character-based, and word boundaries are not marked by spaces. A single Chinese character can be a word by itself, or can be a morpheme of different multi-character words when combined with other characters. Chinese words do not have inflectional markers to specify various types of grammatical properties. Furthermore, many words in Chinese are polysemous, and their meanings have to be fully established on the basis of context by invoking world knowledge or pragmatic information (Zang et al., 2011). To resolve these ambiguities in relation to word boundaries, grammars and meanings, Chinese readers may adopt a different reading strategy (for example, in a delayed rather than an immediate manner) compared with English readers to process pragmatic information. Note, that in a few studies testing this topic on Chinese, the reader relies heavily on contextual information and requires to consult a broad context when facing a local mismatch in a sentence (Zhou et al., 2010; Bornkessel-Schlesewsky et al., 2011; Zang et al., 2011; Jiang and Zhou, 2012; Gibson and Wu, 2013; Jäger et al., 2015). Therefore, a further investigation on Chinese is informative regarding the time point when the pragmatic information is taken into account during sentence comprehension.

Here we investigate when the pragmatic information is used (activated) during reading Chinese sentences of *lian*…*dou*…construction (similar to *even* in English). The *lian*…*dou*… construction normally describes an event which is unlikely to occur but occurred, or an event that is highly likely to occur but did not occur, thus constraining an event of low expectedness. This construction also introduces a pragmatic scale indicating that the focused element (here the embedded event) should lie at the lowest end of the scale and that any event with a higher likelihood than the embedded event must occur. If the event does not rank at the lowest end of the scale, embedding such an event in the construction would result in infelicitousness (Fauconnier, 1975; Yuan, 2006).

Using the event-related potential (ERP) technique with a RSVP, Jiang et al. (2013a) investigated the time course of readers’ usage of the pragmatic information of “unexpectedness” while reading sentences with the *lian*…*dou*… construction. The sentences were presented visually in a word-by-word fashion with each word sequentially presented at the center of the screen with a fixed stimulus-onset-asynchrony (SOA = 800 ms: 400 ms for the word duration and 400 ms for the blank screen). They manipulated the congruence between the constraint of the construction and the likelihood of the event by embedding a highly likely event (incongruent: *Even such a loud sound can he hear clearly, his hearing is so sharp*) or an unspecified event in the sentence (*Even such a sound can he hear clearly, his hearing is so sharp*). They compared both types of sentences to the sentence with a low expected event (congruent: *even such a tiny sound can he hear clearly, his hearing is so sharp*) embedded in the construction. ERPs on the critical verb phrases (VP) showed an increased N400 response in both the incongruent and underspecified conditions, as compared to the one in congruent condition. In the incongruent condition, a slightly larger N400 was elicited as compared to the one in the underspecified condition. Moreover, on the post-critical regions which served as commenting phrase and facilitated the reading of the implied meaning of the *even* constraint – “it is unlikely to hear clearly such a sound,” a sustained negativity was observed in the incongruent condition compared with the congruent and the underspecified conditions, which did not differ between the two. Based on these findings, Jiang et al. (2013a) claimed that Chinese readers can rapidly integrate the critical word or phrase (i.e., the VP) into the pragmatic context, subsequently allowing the observation of the inverse correlation of N400 response with the perceived event likelihood.

These observations are crucial to understanding how readers use the pragmatic constraint information (e.g., event likelihood) to build up sentence representations in Chinese reading (see also Li et al., 2014). From a methodological perspective, the time course of pragmatic processing may be discounted when word-by-word RSVP paradigm is used to study reading. As mentioned earlier, in RSVP, the oculomotor activities are usually restricted, and the preprocessing of the critical VP in the parafovea zone is prevented. Moreover, readers is not allowed to look back and reread earlier parts of the text from which the processing difficulty is re-encountered at a later stage (e.g., on the commenting phrase). This paradigm usually uses a fixed presentation rate which does not always meet natural reading paces that vary across individuals. Hence, findings from Jiang et al. (2013a) may not provide a clear picture of how early the pragmatic information is retrieved and integrated during natural reading activities where the recording of free oculomotor activities is possible.

In the current study, we used eye tracking to examine the exact time course of on-line processing of pragmatic information during natural Chinese sentence reading. Using the same set of sentence stimuli from Jiang et al. (2013a), we constructed congruent, underspecified, or incongruent sentences by embedding less likely, unspecified and neutral, or highly likely events in the *lian*…*dou*…construction (Table 1), and compared the eye-movement patterns in reading incongruent and underspecified sentences with the ones in reading congruent sentences.

**TABLE 1:** An example of a set of sentences used in the experiment.

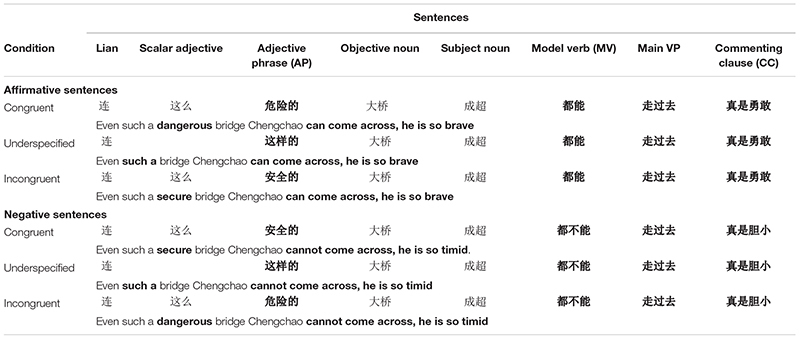

*Regions of interest were bolded.*

To achieve the goal of examining the time course of processing the constraint of event likelihood, all sentences adopted the structure as “*lian* + determiner phrase + object noun + subject noun + *dou* + modal verb (MV) + main verb phrase (VP) + sentence-final commenting clause.” The reader cannot determine the actual event likelihood until he or she reaches main verb phrase, which makes the VP a critical region where the sentence congruency can be detected. Sentence types were varied by manipulating the determiner phrase prior to VP, allowing the observation of any earlier effect before the critical region.

Two accounts may predict differential temporal courses of the use of pragmatic information and differential eye-movement patterns. The “early use” account assumes (Hagoort and Van Berkum, 2007) that the pragmatic constraint (e.g., the unexpectedness that is implied in *lian*…*dou*) is retrieved immediately whenever the building of a representation of event likelihood is possible (e.g., the encountering of VP), and the integration of the linguistic input into the pragmatic constraint takes place without delay. Therefore, an immediate processing cost would be manifested for incongruent sentences, such that readers may experience difficulties as soon as they encounter the VP during first-pass reading; On VP, longer first pass reading time would be expected in the incongruent condition, compared with the one in the congruent condition on the VP region, or even on the pre-VP region such that the effect of event likelihood may be apparent during parafoveal processing (as in Murray, 1998, 2006; Murray and Rowan, 1998). Alternatively, the “late use” account assumes that construction-based pragmatic information is not retrieved immediately when the linguistic input allows the construction of representation for event likelihood and the integration of linguistic input with the pragmatic constraint may induce some delay. Therefore, the incongruency effect could occur in late measures such as the second-pass reading on the critical region or could be more delayed onto the post-critical region (e.g., the commenting phrase). Finally, in the underspecified condition, readers may make pragmatic inference regarding the likelihood of event based on their world knowledge and the pragmatic constraints of the *lian*…*dou*… construction, or engage delayed efforts to integrate an “intermediate” level of event likelihood into the pragmatic constraint (Jiang et al., 2013a; Li et al., 2014). Thus we may observe the modulation of event specification on a second-pass process for the underspecified vs. the congruent condition.

## Materials and Methods

### Participants

Thirty students (12 males and 18 females; Age: 20–26 years, *M* = 22 years; Years of education: 14–17 years, *M* = 15 years) from Tianjin Normal University participated in the experiment. All participants were native speakers of Chinese with normal or corrected to normal vision. None of them reported any neurological, psychiatric, or reading-related disorders. Informed consent was obtained from each participant before the experiment. The study was in accordance with the Declaration of Helsinski and was approved by the Academic Committee of the Academy of Psychology and Behaviour, Tianjin Normal University.

### Apparatus

Participants’ eye movements were binocularly monitored using Eyelink 2000 system (SR Research Ltd., Ottawa, ON, Canada) at a sampling rate of 1000 Hz. Only eye movements of the right eye were analyzed. Sentences were presented on a 17-inch SUMSUNG SyncMaster 959NF monitor with a 1,024 × 768 pixel resolution and a refresh rate of 120 Hz. Stimuli were presented in black on white background in *Song* font. Each character was approximately 28 × 28 pixels in size. The viewing distance was 68 cm, and at this distance each Chinese character subtended approximately 0.92 degrees of visual angle.

### Design and Materials

One hundred and fourteen sets of sentences with *lian*…*dou*… construction were selected from the original stimuli pool developed and used in Jiang et al. (2013a). All sentences were structured in the form of “*lian* + determiner phrase (DP) + object noun + subject noun + *dou* + modal verb (MV) + main verb phrase (VP) + commenting clause (CC)” (see Table 1). Grammatically, the *lian*…*dou*… construction relocates the object noun to an earlier position in the sentence. The *Lian*…*dou*… construction in different experimental sets constrained a different event.

The main VP consisted of an action verb and a verb complement. The embedded event was manipulated by varying the DP, such that the DP was either a scalar adjective phrase “zheme/name/ruci [so] + adjective” to specify the likelihood of the event in the congruent and incongruent conditions or a demonstrative modifier “zheyangde/nayangde/rucide [such]” in the underspecified condition. In each set, the MV was in either affirmative or negative form, with a negation marker *bu* (not) either absent or present immediately before the main VP, creating the affirmative and the negative version of the sentences. Specifically, we replaced the affirmative modal verb with a negative counterpart and switched the adjectives in the congruent and incongruent conditions in the affirmative version to the opposite counterparts in the negative version. The purpose of using the negation form of the stimuli was to prevent readers from expecting the congruence of the sentence based on contextual information preceding the main VP.^1^ Consequently, six sentences were constructed for each set of stimuli.

Both the global sentence comprehensibility (with *lian*…*dou* construction) and the likelihood of an embedded event happening in daily life (without *lian*…*dou* construction) were rated on 7-point scales for each sentence by two independent groups of readers in two off-line tests (see Jiang et al., 2013a for details). Among the stimuli selected for the current experiment, the comprehensibility score was the highest for the congruent sentences (Mean = 6.19, *SD* = 1.45 out of 5, 1- least comprehensible, 7- most comprehensible), lower for the underspecified sentences (Mean = 5.72, *SD* = 1.71), and the lowest for the incongruent sentences (Mean = 2.62, *SD* = 1.82). Reversely, the event likelihood was the highest for the incongruent sentences (Mean = 5.76, *SD* = 0.92 out of 5, 1-least likely, 5- most likely), lower for the underspecified sentences (Mean = 4.46, *SD* = 0.83) and the lowest for the congruent sentences (Mean = 2.48, *SD* = 1.13).

All stimuli were divided into six lists, with each containing 114 formal sentences. Conditions were rotated across lists according to a Latin-square procedure, such that a sentence within a given set appeared only once in each list, and there were equal numbers of sentences per condition per list. In addition, ninety narrative sentences with a canonical structure “Subject noun + Verb + Object noun” were added to each list as fillers to prevent readers from using specific reading strategies generated from certain constructions. In total, each participant was shown a total of 204 sentences which were randomized within the list. Each participant was randomly assigned a list. Ten practice sentences were included at the beginning of each testing session. Among the practice sentences, six had the *lian*…*dou*… structure, and the other four were narrative sentences without the *lian*…*dou*… structure.

In each list, seventy sentences, including 40 critical sentences with the *lian*…*dou*… structure and 30 filler sentences, were randomly selected and followed by a verification statement which required the reader to respond with a yes/no answer. In the 40 verification statements corresponding to the critical sentences, 25 required integrative comprehension of sentential meaning in order for a participant to provide a correct answer. The remaining 15 required information from a specific sentence constituents of the critical sentences, with 9 statements related to adjective phrases, 3 to VP, and 3 to object nouns. Statements concerning the filler sentences were also targeted the meanings of either the whole sentences or specific sentence constituents in different sentential positions. In this way, we made sure that the participants should have read and comprehended the whole sentences before they responded to the verification statements.

### Data Analysis

Several regions-of-interest were predefined for the analysis (see Table 1 for exemplar sentence). The main VP area (e.g., 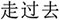, meaning *come across*) was defined as the critical region, where the congruency of the sentence became apparent. The adjective phrase (AP, e.g., 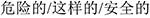, meaning *dangerous/such/secure*) and the model verb (MV, e.g., 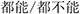, meaning *can/cannot*) were defined as the two pre-critical regions. The MV was defined to detect any possible parafoveal effect on VP that is modulated by the congruency of the sentence. The AP was defined to examine possible regressive saccades into this region due to pragmatic inferences about the likelihood of an unspecified event against the constraints of the *lian*…*dou*… and the integration of specified event into the construction that take place on critical VP. Lexical features of AP were measured and controlled across the three conditions. The number of strokes of AP region was similar across the three conditions (all *M* = 16, *ps* > 0.05), and mean frequencies of this region were higher in the underspecified condition (1072/million) than those in the other two conditions (collapsing congruent and incongruent condition: 146/million, *p* < 0.001). The remainder of the sentence that follows the critical region – the commenting clause (e.g., 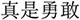, meaning *he is so brave*) was defined as the post-critical region, making the “unexpectedness” meaning explicit.

We computed different eye movement measures that represent different processing stages. The measures for early processing include *first fixation duration* (FFD, the duration of the first fixation on a region during the first pass reading) and *gaze duration* (GD, the sum of all fixations on a region before moving to another region). The measures for late processing include *total fixation duration* (TFD, the sum of all fixations that take place on a region). Moreover, to investigate how participants attempted saccadic movement to deal with processing difficulty due to incongruence or under-specification, the probability of making a *regression in* (REG-IN, regressive saccades from the following regions land into the current region) was reported for pre-critical regions and the probability of making a *regression out* (REG-OUT, saccades departing out of the current region and landing in a previous region, i.e., the interested area at a previous region) was reported for critical and post-critical regions. These two measures indicated the proportion of trials in which a participant made a regressive saccade into/out of a region.

All statistical analysis was performed with linear mixed models (LMM), using the *lme4* package (version 1.1-7) in R (R Core Team, 2014). For all measures per region, we fitted LMM with the maximal random effects structure (Barr et al., 2013), which included both random intercepts and random slopes for the fixed effects over both participants and items. Given that our hypothesis was centered on the effect of pragmatic incongruence and underspecification on eye-movement measures, two contrasts were programed: the first contrast compared the incongruent with the congruent condition to test the “incongruence” effect, and the second contrast compared the underspecified with the congruent condition to test the “underspecification” effect. The congruent condition was treated as baseline in both contrasts to estimate statistical parameters. To reduce the impact of data skewness and facilitate interpretation, all fixation duration measures were analyzed using log-transformed data, and probabilities of regressions were analyzed using logit-link function.

### Procedure

Participants were instructed to read sentences in a normal way that ensured comprehension. They were informed that a simple statement would occasionally appear after a sentence, and they should verify whether the statement was consistent with the message conveyed in the critical sentence by pressing a button on the response box. Prior to the experiment, participants were required to complete a three-point horizontal calibration procedure, with an average calibration error below 0.30 degrees. Prior to the start of each trial, a fixation point was presented on the left side of the screen at reader’s eye-level. Once a stable fixation was detected by the eye tracker, the sentence was presented with the first character replacing the fixation point. Once participants finished reading a sentence, they pressed a response key on a button box to terminate the display. The experiment lasted approximately 50 minutes.

## Results

There was no difference in percentages of correctly responded verification statements corresponding to different critical conditions and the overall mean accuracy was 90%, suggesting that the participants in general read and understood the sentences. Trials were removed from analysis if the track was lost or the total number of fixations were less than 5 (about 0.2% of the total number of trials); additional trials were removed from analysis if an eye movement measure was beyond three standard deviations from the participants’ mean (for the critical region, 0.9%; for the pre-critical region, 1.0%; for the post-critical region, 1.1% of the total number of trials). Among all fixations of all trials, fixation durations shorter than 80 ms or longer than 1200 ms were excluded from the analyses as well. Table 2 shows the means and standard deviations of each eye movement measure per regions of interest. Tables 3, 4 illustrate the statistical estimates of the fixed effects in the LMM for each of these measures.

**TABLE 2:** Eye movement measures for regions of interest, including adjective phrase (AP), dou + modal verb (MV), the main VP and commenting clause (CC) areas.

**Measure**	**Congruent**	**Underspecified**	**Incongruent**
**Pre-critical region 1 – Adjective phrase (AP)**			
FFD (ms)	224(79)	233(83)	222(78)
GD (ms)	288(151)	337(183)	279(146)
TFD (ms)	558(339)	635(388)	643(423)
REG-IN (probability)	0.57(0.50)	0.70(0.46)	0.63(0.48)
**Pre-critical region 2 – Dou + modal verb (MV)**			
FFD (ms)	252(88)	244(82)	249(88)
GD (ms)	324(168)	319(167)	325(172)
TFD (ms)	491(285)	513(316)	553(334)
REG-IN (probability)	0.32(0.47)	0.32(0.47)	0.38(0.49)
**Critical region – Main VP**			
REG-OUT (probability)	0.25(0.43)	0.24(0.43)	0.29(0.45)
FFD (ms)	255(95)	256(96)	256(94)
GD (ms)	354(193)	354(196)	349(191)
TFD (ms)	512(317)	524(343)	548(348)
**Post-critical region *–* Commenting clause (CC)**			
REG-OUT (probability)	0.78(0.42)	0.81(0.39)	0.84(0.37)
FFD (ms)	284(123)	285(127)	291(126)
GD (ms)	436(235)	436(250)	440(230)
TFD (ms)	547(307)	559(327)	608(323)

*FFD, first fixation duration (ms); GD, gaze duration (ms); TFD, total fixation duration (ms); REG-OUT (probability). Probability of regressions-in, i.e., the proportion of regressive saccades on a region from a region with higher index; REG-OUT (probability), Probability of regressions-out, i.e., the proportion of regressing out of a region, limited to the first pass reading of that region.*

**TABLE 3:** Fixed effect estimates for the eye movement measures across pre-critical regions including adjective phrase (AP) and modal verbs (MV).

**Effect**	**FFD**			**GD**			**TFD**			**REG-IN**		
	***b***	***SE***	***t***	***b***	***SE***	***t***	***b***	***SE***	***t***	***b***	***SE***	***z***
**Pre-critical region 1 *–* Adjective phrase (AP)**												
Congruent vs. Underspecified	0.03	0.02	1.39	0.12	0.04	**3.34**	0.13	0.04	**3.19**	0.82	0.20	**4.21**
Congruent vs. Incongruent	–0.01	0.01	–0.68	–0.03	0.02	–1.49	0.12	0.03	**3.70**	0.28	0.11	**2.55**
**Pre-critical region 2 – Dou + modal verb (MV)**												
Congruent vs. Underspecified	–0.03	0.01	−**2.14**	–0.02	0.02	–0.79	0.03	0.02	1.17	0.01	0.10	0.08
Congruent vs. Incongruent	–0.01	0.01	–0.83	0.00	0.02	0.06	0.10	0.02	**4.31**	0.30	0.10	**2.87**

*Significant terms are marked in bold. *b*, regression coefficient.*

**TABLE 4:** Fixed effect estimates for the eye movement measures across critical and post-critical regions including main VP and commenting clause (CC).

**Effect**	**REG-OUT**			**FFD**			**GD**			**TFD**		
	***b***	***SE***	***z***	***b***	***SE***	***t***	***b***	***SE***	***t***	***b***	***SE***	***t***
**Critical region *–* Main VP**												
Congruent vs. Underspecified	0.07	0.14	0.50	0.00	0.02	0.05	0.00	0.02	0.08	0.01	0.03	0.24
Congruent vs. Incongruent	0.32	0.16	**2.01**	0.01	0.02	0.34	−0.01	0.02	−0.35	0.06	0.03	**2.07**
**Post-critical region-commenting clause (CC)**												
Congruent vs. Underspecified	0.37	0.16	**2.38**	0.00	0.02	0.15	−0.01	0.02	−0.52	0.01	0.03	0.41
Congruent vs. Incongruent	0.68	0.20	**3.32**	0.02	0.02	1.01	0.00	0.02	0.16	0.10	0.03	**3.22**

*Significant terms are marked in bold. *b*, regression coefficient.*

### Pre-critical Region 1 – Adjective Phrase (AP)

Readers spent longer time fixating on the AP region in the underspecified condition than in the congruent condition during the first-pass reading (GD: *b* = 0.12, *SE* = 0.04, *t* = 3.34). There was no significant difference in first-pass reading time between congruent and incongruent conditions. This result suggests that readers were requested more time to parse the sentence with underspecified adjective phrase while they encountered no increased cost for the incongruent sentences during the first-pass reading.

However, for the total fixation duration, readers spent longer time fixating on the AP region when reading the underspecified and incongruent sentences, as compared to reading the congruent sentences (Underspecified vs. Congruent, *b* = 0.13, *SE* = 0.04, *t* = 3.19; Incongruent vs. Congruent, *b* = 0.12, *SE* = 0.03, *t* = 3.70). Furthermore, with more linguistic information accumulated for the underspecified and incongruent conditions, the readers were more likely to make regressions back to the pre-critical region (Underspecified vs. Congruent, *b* = 0.82, *SE* = 0.20, *z* = 4.21; Incongruent vs. Congruent, *b* = 0.28, *SE* = 0.11, *z* = 2.55).

### Pre-critical Region 2 – Model Verb (MV)

The measures on MV may reflect parafoveal congruency effect on the critical VP prior to the fixation. Readers spent shorter first fixations on the MV region in the underspecified sentences than in the congruent ones (FFD: *b* = −0.03, *SE* = 0.01, *t* = −2.14). This reduced FFD on the MV in the underspecified condition might be due to the increased FFD in the same condition on the earlier AP region. The readers may initiate the inference of missing scalar adjectives based on their knowledge or pragmatic constraints of the *lian*…*dou*… construction to deal with the uncertainty of event likelihood in the underspecified sentences. With the initial missing scalar adjectives filled, it may cost less to process the upcoming MV during the first pass reading.

However, later measures showed longer TFD and more REG-IN in the incongruent relative to the congruent sentences (TFD: *b* = 0.10, *SE* = 0.02, *t* = 4.31; REG-IN: *b* = 0.30, *SE* = 0.10, *z* = 2.87). These results suggest that the processing difficulty for the incongruent condition did not appear as an early parafoveal processing mechanism prior to the fixation. The incongruent condition did not affect the initial processing of MV, but the later measures, probably involving re-checking linguistic information of event likelihood at earlier regions after the incongruency, has been detected in the later regions.

### Critical Region – Verb Phrase (VP)

None of the first-pass reading time measures (including FFD and GD) showed any significant effects of incongruency or underspecification (all *p*s > 0.05). However, readers spent longer total fixations on and made more regressive saccades out of VP in the incongruent condition than they did in the congruent condition (TFD: *b* = 0.06, *SE* = 0.03, *t* = 2.07; REG-OUT: *b* = 0.32, *SE* = 0.16, *z* = 2.01). This suggested that readers did not encounter any difficulties or initiate any effort to deal with the difficulties immediately after detecting the infelicitous nature of the main clause. The incongruent sentence exhibited prolonged reading time in the later measure of main VP of incongruent sentences.

### Post-critical Region – Commenting Clause (CC)

Similar to the findings on VP, readers spent longer TFDs on CC in the incongruent condition than in the congruent condition (*b* = 0.10, *SE* = 0.03, *t* = 3.22). Furthermore, there were significantly more regressive saccades out of the CC region back to previous regions in the incongruent and underspecified conditions than in the congruent condition (Incongruent vs. Congruent, *b* = 0.68, *SE* = 0.20, *z* = 3.32; Underspecified vs. Congruent, *b* = 0.37, *SE* = 0.16, *z* = 2.38). These data suggested that the incongruent pragmatic information did not result in the lengthening of the initial reading time but only prolonged the global reading time at the sentence-final clause. No other effects were significant for FFD and GD on CC (all *p*s > 0.05).

## Discussion

Using the same set of sentence stimuli as the previous study (Jiang et al., 2013a) and taking advantage of the eye-tracking technique, we re-visited the temporal course of processing the construction-based pragmatic constraint (i.e., the event likelihood) during natural Chinese sentence reading. We obtained novel evidence on sentences with *lian*…*dou*…construction (similar to *even* in English) in which the likelihood of the embedded event to occur was manipulated. By embedding a highly likely or an underspecified event in the sentence, we created the incongruent and the underspecified conditions, and compared each with congruent sentences in which an unexpected event was embedded.

Unlike the previous ERP study (Jiang et al., 2013a) in which the segmented words were presented sequentially and separately, the current study allowed readers to make saccadic movements back and forth spontaneously. Moreover, the sentence comprehension was minimally demanded with a task to probe readers by answering questions about the sentence (cf. Jiang et al., 2013a), creating an opportunity to examine an implicit use of pragmatic information during sentence reading. We will discuss how these methodological factors contribute to the eye movement activities later.

In addition to the eye-movement measures on critical regions (“VP”) where the nature of condition (incongruent or unspecified) was determined, we calculated such measures on regions prior to or following the VP. Depending on experimental conditions, these regions were hypothesized to attract more or less saccadic looks given the necessity to specify eventual representations (such as on “AP”), given the possible parafoveal views on the critical region that permits an early detection of pragmatic constraints (such as on “MV”), or given the possible wrap-up process for the whole discourse (such as on “CC”). On the pre-critical regions (particularly the MV region), there was no evidence indicating that the incongruent information can be processed parafoveally. Consistent with previous studies (Ni et al., 1998; Rayner et al., 2004; Filik, 2008; Filik et al., 2009), there was no significant effect of pragmatic congruence on the first-pass reading time of the critical region, where the incongruity of a sentence became apparent. However, readers did spend longer total reading time, and made more regressive saccades out of the VP region to the pre-critical regions in the incongruent condition, as compared to the congruent condition. Similar observations were also made in the post-critical region. These results suggest that there was no immediate processing cost associated with the reading of pragmatically incongruent information relative to the reading of congruent information. When the event likelihood is unspecified, the effort of rereading and regressive looks were requested to a far lesser extent than a sentence with an incongruent event, as the differences between these conditions were only obvious on the late measures of sentence-final region (see later for section “Discussion”). The differences in the first-pass reading on AP does not seem to be driven by lexical features (e.g., word frequency); the early reading time seems to increase when the linguistic information specifying the event likelihood is absent in the underspecified condition.

Overall, the critical findings in relation to the comparison between pragmatically incongruent and congruent sentences clearly indicate that interruption of the integration of event likelihood into the pragmatic constraints of the *lian*…*dou*… construction does not intervene with the eye movement measures immediately as the information about the event likelihood becomes salient. Our results are comparable with some of the previous research investigating the effects of pragmatic implausibility (e.g., Ni et al., 1998; Braze et al., 2002; Rayner et al., 2004; see Warren, 2011 for a review) on eye movements in reading. For example, Rayner et al. (2004) found that when a word was completely anomalous in a context or against one’s real-world knowledge, increased gaze duration can be observed on the anomalous word without delay. However, when a word was implausible but still possible to appear in the sentence, the so called “pragmatic anomaly,” the effect did not emerge until a considerably later time – *go-past* time. Our results also extended the findings of Filik et al. (2009), that the effect of incongruence in sentences with the *even* construction was not evident until a post critical region, to a language other than English. Presumably, these measures suggest that the increased difficulty is initiated by some sort of second-pass processing in search of more information to resolve the incongruence between the current event and pragmatic constraints. When processing *lian*…*dou*, to check whether the event indeed fits the lowest end of the pragmatic scale, readers need to contrast a particular event against a set of alternatives on the event likelihood scale, and decide whether this event can be an unexpected candidate or sits at the bottom of the scale. This difficulty was increased given the mismatch of the linguistic input and the prediction of the *lian*…*dou* constraint. Therefore, readers spent more time to recover from this mismatch and probably recheck any further information to resolve such mismatch (Jiang et al., 2013a), resulting in more regression-in on the pre-critical region and regression-outs on the critical/post-critical regions. Increased regressive saccades were reported for sentences with long distance dependencies which demand higher working memory load (e.g., in *who does Mary think that John calls?* Nicenboim et al., 2015). Here the AP, the key linguistic information that defines the event likelihood, is possibly reactivated on regions following AP and may demand higher working memory load as reflected by more regressive looks to reconfigure the event likelihood in the incongruent condition. The increased reading time on the sentence-final commenting phrase suggested a continued difficulty that arose earlier from the critical VP. This sentence wrap-up effect was consistent with the observation of an increased sustained negativity on that phrase in Jiang et al. (2013a). The pragmatically implausible word increased the rereading time (i.e., total reading time minus gaze duration) and probability of regression-out when it was located at the sentence-final position (Camblin et al., 2007a). It should be noted that the underspecified condition did not show any effect on VP but showed more regression out of the sentence-final position, possibly due to an effort to wrap up the sentence by rechecking previous AP (as reflected by increased regression-ins on AP) against the possibility of specifying the meaning of the event (Zhou et al., 2010; Jiang and Zhou, 2012; Jiang et al., 2013a).

### Implications to Models of Pragmatic Processing

Our findings appear to contradict the ERP results (Jiang et al., 2013a) which argue for a “one-step” model of pragmatic processing (Hagoort and Van Berkum, 2007). The eye-tracking data cannot be accommodated easily by the “one-step” but may fit into a “two-step” language processing model. According to the latter model, in the first step, the local, context-independent meaning of a local structure is computed; only when this step is completed, the meaning is computed against the wider sentential, discourse and communicative context or against an individual’s pragmatic knowledge (Grice, 1975; Fodor, 1983; Sperber and Wilson, 1995; Cutler and Clifton, 1999; Lattner and Friederici, 2003). This model is in contrast with the “one-step” model which assumes that different levels of meanings are activated simultaneously in the context, resulting in a unified N400 on words in ERPs that mismatched a diverse set of contextual information (Hagoort and Van Berkum, 2007), including the N400 effect on VP in the incongruent condition in Jiang et al. (2013a). Given that N400 typically indexes the immediate impact of pragmatic constraint during online linguistic processing (Kutas and Federmeier, 2011), it was concluded that the pragmatic information is rapidly used in online sentence reading.

The current data that tracked readers’ eye-movement do not fully agree with the conclusion above. In the *lian*…*dou* construction, the reader has to form the representation of the event based on the local structure “determiner phrase + object noun + subject noun + VP,” of which the likelihood is reversed by *lian*…*dou* in the global context. The “one-step” model would predict that pragmatic constraints of *lian*…*dou* is used in an immediate manner; this prediction was rejected by the lack of early modulation of congruency manipulation. In contrast, the specification of local event likelihood was manifested as an increased first-pass fixation duration in the underspecified condition, suggesting that the buildup of a local semantic meaning *can* be early. The *lian*…*dou* constraints are taken into account only when local representation is partially built and may be reanalyzed through initiating regressive saccades to the preceding sentential constituents whenever necessary.

The two-stage processing is consistent with recently proposed eye movement control models. For example, the E-Z Reader 10 (Reichle et al., 2009; see Reichle, 2011 for a review) specifies when the higher-level, post-lexical information affects eye movements during language comprehension. The model assumes that integration of a word into its syntactic and semantic context comes after the process of word identification, which is therefore post-lexical. Staub and colleagues (Staub, 2011; Abbott and Staub, 2015) provided evidence supporting this assumption as they observed that the integration difficulty of an implausible word (e.g., *the professor repaired the writer with a trusty old wrench*) does not appear on the early measures on the critical word (e.g., the skipping rate of *writer*) but appears downstream of that word. Even though the plausibility effect can, in some cases, be manifested in the first-pass fixation measures on a target word (Staub et al., 2007; Matsuki et al., 2011), the plausibility and other lexical effects (e.g., word frequency) are typically additive, suggesting the pragmatic information may not impact local processing in the early time course during sentence reading (Abbott and Staub, 2015). These model-guided experimental findings suggest that computation of plausibility or higher-level pragmatic meaning affects post-lexical integration, instead of lexical identification itself, during sentence comprehension.

How can we reconcile the contradictory findings between Jiang et al. (2013a) and the current study? In Jiang et al.’s study, each word (or phrase) was presented serially for 400 ms followed by an inter-stimulus interval (ISI) of 400 ms. Previous studies have shown that the presentation rate may affect the manifestation of different cognitive processes: the contextual effect is more likely to emerge without delay in a prolonged presentation rate (Camblin et al., 2007b). Similarly, the comparatively slower RSVP rates of word presentation in Jiang et al. (2013a) may provide readers with sufficient time to integrate the critical VP with the pragmatic information conveyed by *lian*…*dou*, allowing the effect of congruence-related N400 to appear on the VP.

In the current eye-tracking paradigm, sentences were presented as an entirety in one line, and the readers were allowed to preview information and initiate regressive saccades to reanalyze uncertain or incongruent linguistic input. In an ERP study when readers were allowed to read at their own pace, longer reading time was predicted by larger amplitudes of ERP on words mismatching pragmatic constraint (e.g., less plausible sentence: *at the breakfast the boy would plant toast and jam*, Ditman et al., 2007), indicating that the immediacy of pragmatic congruency is affected by presentation speed. Moreover, in a task that does not emphasize the verification of acceptability of the sentence (cf. Jiang et al., 2013a), it is likely that the reader may adopt a good-enough strategy (Ferreira et al., 2002; Ferreira and Patson, 2007) as the demand of recovering from the pragmatic incongruence during normal sentence reading is low; consequently the incongruence effect appears late.

In summary, by using the eye tracking technique, the present study reveals a relatively delayed time course of processing pragmatic constraints during on-line reading of Chinese sentences with *lian*…*dou*…construction. When reading incongruent sentences, as compared with congruent ones, the reader spends longer total fixations, made more regressive saccades out of the critical regions where pragmatic infelicitousness is initially detected. This finding is comparable to the observation of *even* construction in English (Filik et al., 2009) which showed a delayed processing cost and an effort of reanalysis for highly likely events used after *even*. The current study provides new evidence showing that the processing of pragmatic constraints of the Chinese *lian*…*dou*… construction may not interrupt the early stage of lexical processing during natural sentence reading, and offers a methodological perspective that promotes ecological studies of language processing.

## Data Availability Statement

Original data covered by this study can be obtained from the corresponding authors upon request.

## Ethics Statement

The studies involving human participants were reviewed and approved by the Academic Committee of the Academy of Psychology and Behaviour, Tianjin Normal University. The patients/participants provided their written informed consent to participate in this study.

## Author Contributions

CZ, XB, GY, XJ, and XZ designed the research. CZ, LZ, and MZ performed the research and analyzed the data. CZ, XB, GY, XJ, ZH, and XZ wrote the manuscript.

## Conflict of Interest

The authors declare that the research was conducted in the absence of any commercial or financial relationships that could be construed as a potential conflict of interest.

## Footnotes

1 To examine whether the form of sentence (affirmative vs. negative) could module the key effects of sentence type, we conducted a set of LMM analyses in which the sentence form was included as a fixed factor in addition to the sentence type. These analyses did not produce any reliable interactions between the sentence form and the sentence type for any measures over the two pre-critical regions, critical and post-critical regions (all *ts* < 1.75), indicating that this variable did not contribute significantly to our key findings.
